# CAPE for measuring callous-unemotional traits in disadvantaged families: a cross-sectional validation study

**DOI:** 10.12688/f1000research.19605.2

**Published:** 2020-02-27

**Authors:** Luna C.M. Centifanti, Hannah Shaw, Katherine J. Atherton, Nicholas D. Thomson, Susanne MacLellan, Paul J. Frick

**Affiliations:** 1 Department of Clinical Psychology, University of Liverpool, Liverpool, UK; 2 Department of Psychology, University of Durham, Durham, UK; 3 Department of Surgery Division of Acute Care Surgical Services, Virginia Commonwealth University, Richmond, USA; 4 Department of Psychology, Louisiana State University, Baton Rouge, USA; 5 Institute for Learning Sciences and Teacher Education, Australian Catholic University, Sydney, Australia

**Keywords:** Assessment, Callous-Unemotional Traits, Conduct Problems, Families, Personality, Externalizing Behavior

## Abstract

**Background:** Callous-unemotional (CU) traits are important for designating a distinct subgroup of children and adolescents with behaviour problems.  As a result, CU traits are now used to form the specifier “with Limited Prosocial Emotions” that is part of the diagnostic criteria for the Conduct Disorder in the Diagnostic and Statistical Manual of Mental Disorders 5 ^th^ Edition (DSM-5) and International Classification of Diseases 11 ^th^ Revision (ICD-11).  Given this inclusion in major classification systems, it is important to develop and test methods for assessing these traits that can be used in clinical settings.  The present study aimed to validate a clinician rating of CU traits, the Clinical Assessment of Prosocial Emotions, Version 1.1 (CAPE 1.1), in a sample of hard-to-reach families referred to a government program designed to prevent the development of behaviour problems in high risk families.

**Methods:** Clinical ratings of children were obtained from 34 families of children ages 5 to 18 (M=13.5; SD=3.2). The ratings on the CAPE 1.1 were based on interviews with both parent and child.

**Results** **:** Of the sample, 21% (100% male) met the diagnostic cut-off for the specifier according to the CAPE 1.1, and CAPE 1.1 scores were associated with parent ratings of CU traits, psychopathic traits, and externalising behaviours. CAPE 1.1 ratings were also associated with risk for violence obtained from case files.

**Conclusions:** These findings provide preliminary evidence for the validity of the CAPE 1.1 as clinician rated measure of CU traits.

## Introduction

Callous-unemotional (CU) traits include behaviours that reflect a lack of caring for others and for doing things well, a lack of guilt and remorse, and a lack of emotional depth in interactions with others ^1^. CU traits across childhood and adolescence have been associated with delinquency, aggression and impairing conduct problems ^2– 4^. These traits have proven useful for designating a subgroup of children with serious conduct problems who show distinct emotional, cognitive, and social correlates relative to other youth with behaviour problems ^5^. Further, there is evidence that children with serious behaviour problems who also show elevated levels of CU traits show less positive responses to many interventions used to treat conduct problems ^6, 7^. As a result of this evidence for both their clinical and etiological validity, CU traits have recently been included in the specifier “with Limited Prosocial Emotions” (LPE) for the diagnostic criteria for Conduct Disorder in the DSM 5 (Diagnostic and Statistical Manual of Mental Disorders, 5th Edition; ^8^) and for the diagnostic criteria for Oppositional Defiant Disorder and Conduct Disorder in the ICD-11 (International Classification of Diseases version 11; ^9^).

Conduct problems are one of the most frequent reasons that youth are referred for mental health treatment ^10^ and they are very costly to the community due to their association with delinquency ^11^. As noted above, those children with serious conduct problems who also have elevated CU traits show less positive responses to many forms of treatment, possibly because of their deficits in emotional responding to the distress in others and abnormalities in their reward and punishment processing ^12– 14^. However, certain treatments that are tailored to their unique emotional and cognitive style have shown some limited success in children and adolescents with elevated CU traits ^7, 15, 16^. Thus, accurate assessment of these traits is critical for treatment planning ^17^.

With the inclusion in major classification systems used to diagnose childhood behaviour problems and their importance for guiding effective treatment, it is crucial to have measures of CU traits that can be used in high risk samples to make important clinical decisions. This is especially true when children are young, since early interventions tend to be most effective ^18– 20^. To date, much of the research on CU traits has relied on informant rating scales that have proven to reliably assess these traits from the age of three years ^16, 21^ and in a way that is relatively stable between childhood and adolescence ^22, 23^. Further, behaviour ratings provide a very time-efficient method for assessing these traits in research because they are easy to complete and do not require high levels of training to administer and interpret.

However, there are significant limitations in the sole reliance on rating scales for making clinical decisions. First, well-established clinical cut-offs are not available for many of these measures to designate when the level of these traits is severe and impairing enough to warrant a diagnosis ^21, 24^. Second, as is the case for all forms of psychopathology in children ^25^, ratings of CU traits from different informants often show only modest levels of agreement ^26^. This lack of correspondence across different sources of information makes it a challenge for clinicians to know how best to integrate these discrepant reports when making diagnoses ^27^. Third, a number of biases, both intentional (e.g., deception) and unintentional (e.g., social desirability), can influence ratings of CU traits that need to be considered when making clinical decisions and such sources of bias are often difficult to determine from questionnaires ^27– 29^. Fourth, it appears that the optimum assessment of CU traits that detects the construct across all levels of severity requires the use of both positively worded items in which affirmative response denotes more callous and unemotional behaviours (e.g., “do you seem cold and uncaring to others”), as well as negatively worded items in which an affirmative response denotes lower levels of the construct (e.g., “do you feel bad or guilty when you do something wrong”; ^30^). However, negatively worded items can be difficult to understand and put strain on a child’s verbal abilities, which have been found to be deficient in children with conduct problems ^31^. Thus, when making clinical decisions, it is important that items are accurately understood by those providing information. A final issue with existing rating scale measures of CU traits is that they were not developed specifically to assess the criteria for the LPE specifier included in the DSM-5 and ICD-11, which further complicates their use for making the diagnosis ^21^.

To overcome these limitations of existing measures of CU traits, the Clinical Assessment of Prosocial Emotions, Version 1.1 (CAPE 1.1; ^32^) was developed to assess CU traits in children and adolescents ages 3 to 21 years of age. The CAPE 1.1 was designed specifically to assess the four symptoms of the LPE specifier now included in the DSM 5: lack of guilt/remorse, callousness and lack of empathy, unconcern about performance, and shallow or deficient affect. The CAPE 1.1 uses the structured professional judgement approach to assessment. That is, very explicit descriptions of the four symptoms are provided and experienced clinicians rate the children on each symptom using a three-point scale (0=not at all descriptive; 1=somewhat descriptive; 2=definitely descriptive) based on all sources of information. The manual has descriptions of prototypical behaviour for each of the four items to aid the interviewer in making their ratings. Also, the CAPE 1.1 is explicitly designed to not rely on any single source of information and it provides a semi-structured interview format to aid the clinician in obtaining information from the child, parents, and other informants. These interviews start with stem questions directly related to the items being rated (e.g., “Does ________ seem to care and be concerned about the feelings of others?”). The clinician follows up by asking for examples to ensure that the informant understood the question and by asking other questions (e.g., “Is this how he is most of the time and with most people”) to aid in scoring. The semi-structured format allows the interviewer to probe and ask for as many examples as needed. The final ratings are only made by considering all sources of information and, to conform to DSM-5 criteria, a diagnosis is made when two or more items are scored as being “very descriptive” (the maximum score of 2) of the child.

In summary, the CAPE 1.1 was designed to overcome many of the limitations of existing measures of CU traits for making clinical diagnoses of the LPE specifier. However, to date, there has been no published data testing the validity of this method. Thus, we investigated the validity of the CAPE 1.1 in a group of socially disadvantaged families that are part of the ‘Troubled Families Scheme’ and the ‘Family Intervention Programme’ - implemented by the UK government. Children who show serious conduct problems tend to come from high-risk backgrounds involving disorganized, highly stressed, or economically disadvantaged families ^33^, and the schemes provide early intervention for these behaviour problems facilitated by local county councils. The schemes target families in which parents experience unemployment, their children fail to attend school, and where family members are involved (or at risk of being involved) in drug use and/or other criminal activity. Also, at least one child of the targeted families had been identified by teachers as exhibiting symptoms linked to conduct problems. The stated aim is to break the cycle of disadvantage that leads to antisocial behaviour across generations. We examined the relations among the CAPE 1.1 item scores and criterion-related measures with well-validated questionnaires of CU traits and psychopathic traits in a sample of families in the Troubled Families Scheme. We also validated the CAPE 1.1 with construct-validity measures of offending, and violence from case file records. We examined the relation between the CAPE 1.1 ratings and well-validated questionnaire measures of externalizing (conduct problems) and internalizing behaviours as well as impact of symptoms on daily living. Finally, we examined the validity of the diagnostic cut-off used by the CAPE 1.1 to determine clinical levels of CU traits, as specified in the DSM-5 criteria for the LPE specifier. We decided not to confound the validation measures with CAPE 1.1 ratings, so ratings were solely based on the semi-structured interview.

## Method

### Ethical considerations

Ethical approval was given by the Psychology Ethics Sub-Committee at University of Durham (approval #12-20). Investigators accompanied a county council caseworker to potential participants’ homes. Inclusion criteria were families enrolled in the “Troubled Families Programme” or the “Family Intervention Project”. Participants were identified by the county council as those families who posed very little risk to investigators’ safety. No exclusion criteria were employed. The caseworkers sought permission from families for the investigators to visit to explain the study. At the homes (or in one case, an office), investigators briefed participants and explained that the study was run by Durham University researchers and was separate from the consent process for entering the county council’s programmes. Parents or carers gave written consent to take part for themselves and on behalf of their children. Children were asked for their assent. Families were told all information would be confidential, but exceptions to confidentiality included risk of harm. These families were already in care of the caseworker so the caseworker would have been notified of any risks discovered by investigators and would have notified the police, if necessary. This was never found to be necessary. All investigators held current Disclosure and Barring Service clearance certificates.

### Participants

Participants were families who were registered as one of two government schemes in the North East of England. A total of 34 families took part, which were based on availability. As result, no a priori power analysis was conducted to inform sample size. Of these families, 24 were enrolled as part of the Troubled Families Scheme, and 10 with a closely aligned (managed by the same team of case workers and with the same interventions offered) Family Intervention Project. These groups did not differ on any of the study measures, so we combined the data. Each family had a target child, whereby the child was identified with the most problematic behaviour or was the focus of the intervention. The questionnaires and interviews that formed the evaluation were completed by the mother of the family and the target child. In one case the mother and father of the child completed the questionnaires and interview together and, in another case, these were completed by an older sister who was the legal guardian. Other children in the family home were asked to fill out questionnaires (with the investigator reading all items aloud).

The target child was older on average (M=13.5; SD=3.2; range: 5–18 years) than the non-target children in the same families, typically biological siblings (M=10.9; SD=4.7; range 3–22 years). With regard to gender, 24 (69%) of the 35 target children (one family had two target children) were male, while 25 out of 50 (50%) of the non-targets were male. The difference between the target groups on gender (Χ ^2^= 2.91, *p* = .09) was not significant.

**Figure 1.**
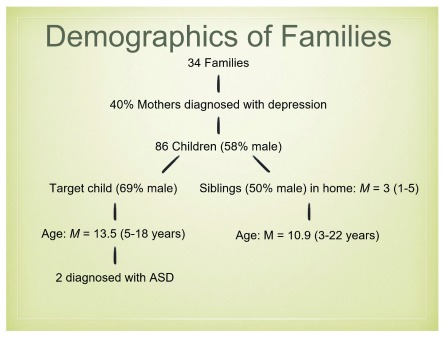
Demographics of families and children selected for participation. ‘Target child’ designates children who were the target of the “troubled families” intervention.

One of the target children was selected at random from the family that had two target children (and who were twins). Therefore, the total sample of children (N=84), consisted of 34 target children and 50 non-target children. See Figure 1 for a flow chart of the selected families and siblings. Only data from the target child will be presented, since they are the only ones who had a completed CAPE.

### Procedure

Families already targeted by the Troubled Families scheme or Family Intervention Programme were contacted by the Stockton Community Safety Team to be invited to take part in this study. They were told that participation was voluntary and that their decision would not affect involvement in the respective scheme. Participants did not receive incentives for participating. We used the judgement of the case workers regarding safety to enter family homes, and we were always accompanied by one case worker for the hour in which we conducted assessments. Only 6 families were deemed unsuitable or declined, within the time frame in which we conducted the assessments (from December 2013 to September 2014). These were unsuitable because either the caseworkers were themselves afraid of violence or the families were already non-compliant with the caseworkers efforts at intervention. Investigators collected data at participants’ homes (except for one case, which was completed in a private room at the county council building). Parents gave written consent to take part for themselves and on behalf of their children; children were verbally asked for assent.

### Measures

Links to all measures used in this study are provided as extended data

**Clinical Assessment of Prosocial Emotions, Version 1.1** (CAPE 1.1; ^32^). The CAPE 1.1 was scored in the current study based on semi-structured interviews conducted with the target child and parent in all but three cases. For two children below the age of 7 years and for a child with a diagnosis of Autism Spectrum Disorder (ASD; and who had severe symptoms), only the parent interview was conducted. The other child with ASD was capable of being interviewed. We decided to do assessments using the CAPE regardless of ASD, because research shows that some children have a ‘double hit’ of ASD and callous-unemotional traits ^34^ and the modest phenotypic overlap observed in social-emotional deficits is largely explained by shared genetic variance ^35^. While the CAPE 1.1 is designed to use all sources of information available to the clinician, including semi-structured interviews, rating scales, and file review, the scoring in the current study was based solely on the interviews, so that rating scales and file information could be used to test the validity of the CAPE 1.1 scores. Clinicians rated the four items of CAPE 1.1 using the 3-points described previously. The CAPE 1.1 was carried out by the first and fourth authors (LCMC and NDT) who, despite not being registered clinicians, had a sufficient level of qualification, clinical training and research experience to carry out the assessments. The manual specifies that raters use their developmental psychology training to ensure that symptoms are rated with regard to what is typical behaviour for a child based on their age. Both raters had sufficient training in developmental psychology and in the assessment of callous-unemotional traits in children as young as 2–3 years of age ^36^. We used scores from the CAPE 1.1 in two ways. First, we used the number of symptoms rated categorically as being “very descriptive”: the number of items rated “2”. Thus, the range was 0 to 4. Second, we examined the prevalence and validity of the diagnostic threshold used by the CAPE 1.1 to approximate the Limited Prosocial Emotions specifier, as defined in the DSM 5 ^8^, which defines those with the diagnosis as persons with 2 or more symptoms rated as 2.

**Child Problematic Traits Inventory (CPTI;** ^37, 38^ **)**. The CPTI is a 28-item measure that was originally developed for teachers to report on psychopathic-like traits for children, but it has been used to obtain parent-reports in one previous investigation ^27^. Thus, we used the parent reports on the total score (α = .93) as well as the three sub-scales measuring CU traits (α = .91), grandiosity/deception (α = .90), and impulsivity/need for stimulation (α = .85), since we were interested in the relations with psychopathic traits in general.

**Strengths and Difficulties Questionnaire (SDQ;** ^39^ **).** The SDQ is a 25-item scale that assesses five domains of adjustment including Hyperactivity, Conduct Problems, Emotional Symptoms, Peer Problems, and Prosocial Behavior. Generally, the SDQ was found to be a reliable and valid measure of conduct problems and has been widely used in both community and clinical samples of children and adolescents ^40^. A three-subscale division has been recommended by Goodman *et al.* ^41^ consisting of externalizing behaviour, internalizing behaviour and the prosocial scale. Externalizing behaviour was assessed by combining the Hyperactivity and Conduct Problems sub-scales, on both a parent (α = 0.89) and child (α = 0.75) version. Internalizing behaviour was also assessed using the child (α = 0.48) and parent (α = 0.76) versions, combining Emotional Symptoms and Peer Problems. The prosocial scale was utilized as part of the University of New South Wales (UNSW) CU Traits measure (see below). In addition, part of the extended version of the SDQ, the impact supplement was used to assess further chronicity, social impairment, distress and burden to others ^42^. This is a scale, based on parent-reports, that sums items about impact on free time and leisure, home activities, and school activities, as well as distress caused by symptoms.

**UNSW CU Traits** ^43^. The UNSW measure of CU traits has been created from items on the prosocial (5 items) and conduct problems (1 item) sub-scales of the SDQ. Additionally, three items from the Antisocial Process Screening Device (APSD; ^44^) are included in this scale. This measure was created based on a factor analytic assessment of the SDQ and APSD and has since been extensively validated ^45, 46^. The 9-item measure was collected using parent (α = 0.87) and child (α = 0.78) reports.

**Case file records.** A dichotomous measure (1=present, 0=not present) was created from the risk assessment for violence that caseworkers assessed as part of their work with the families; a risk assessment of ‘present,’ meant that violence was of concern to caseworkers and they had actions in place to minimize harm to themselves or others. We also created dichotomous variables of the target child’s involvement (1=yes, 0=no) with Young offending services (YOS) as a measure of delinquent behaviour since this service (from the UK’s National Health Service) is concerned with treating and rehabilitating juveniles who have engaged in delinquency.

### Statistical analysis

JASP 0.9.1.0 ^47^ was used for t-tests, correlations, descriptive statistics, and chi-square analyses. Seven of the target youths refused to participate in filling out the questionnaires, so correlations with the CAPE reflect this when using child-report, but parent-report was available. For one of the target children, researcher error meant that the parent only completed the CPTI but not the SDQ.

## Results

### Clinical assessment ratings of Limited Prosocial Emotions

**Figure 2.**
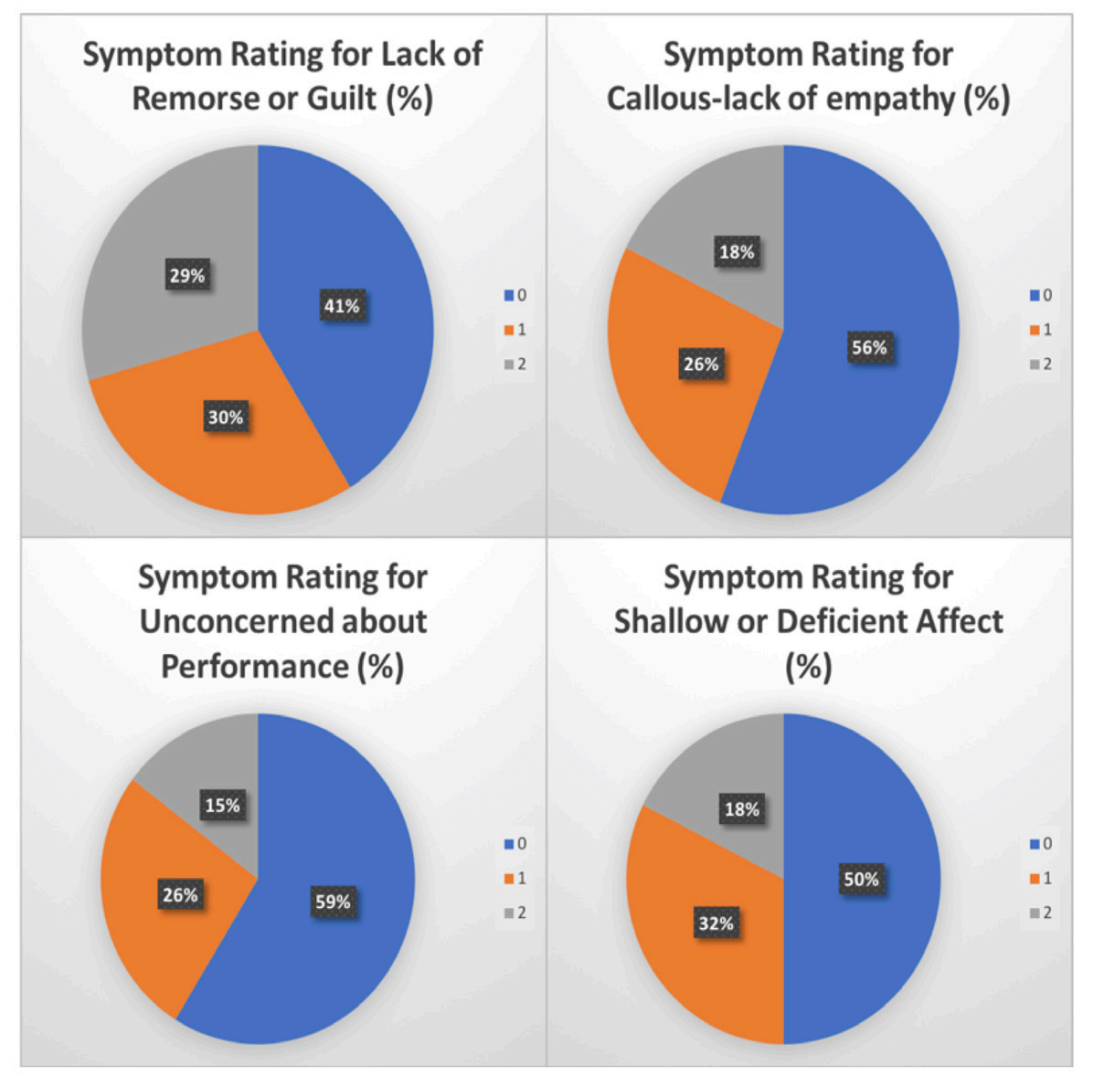
Proportions of target children given ratings of 0 ‘not descriptive or mildly descriptive’, 1 ‘moderately descriptive, and 2 ‘very descriptive’ on the CAPE 1.1.

Figure 2 shows the percentage of target children that scored at each level of severity for each symptom on the CAPE 1.1. The most frequent rating given to children was ‘0’ for all symptoms, ranging from 41% to 59% across the four symptoms. The least frequent rating was the maximum score of “2”, which ranged from 15% to 29% across symptoms. Of the 34 target children, seven children (21%; all males) met diagnostic criteria for the LPE specifier (two or more items were rated at a maximum value of ‘2’). Thus, the majority of the sample did not meet the threshold for the diagnosis, and most did not have symptoms that reached a clinical range.

**Figure 3.**
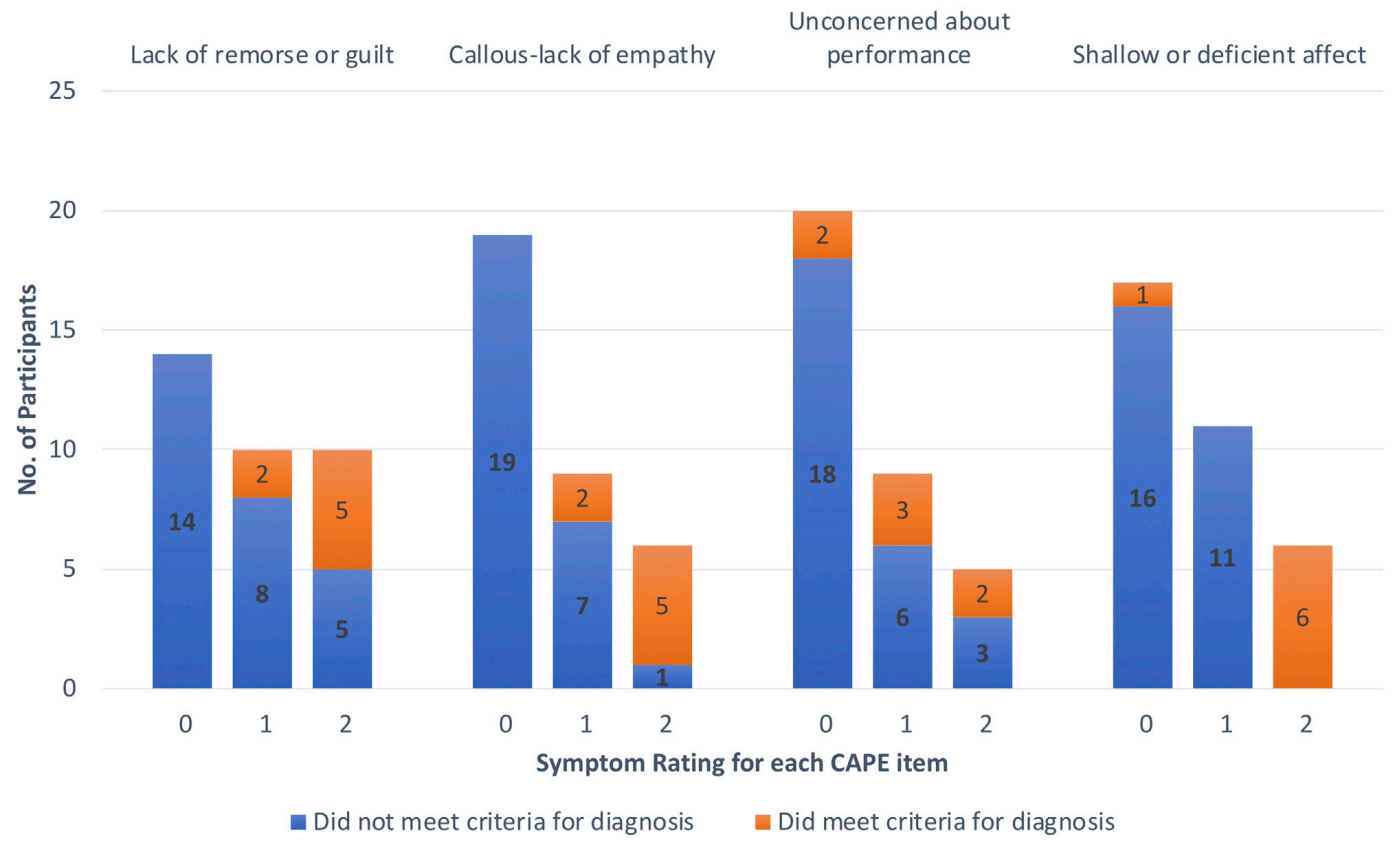
Frequency of use of each CAPE 1.1 rating by CAPE 1.1 item and LPE diagnostic status.

To determine whether all items equally discriminated those meeting diagnostic criteria on the CAPE, we examined the frequency of symptom scores within those who did and not meet the threshold for the LPE specifier. The results are provided in Figure 3. For ‘lack of remorse or guilt’, ‘callous-lack of empathy’ and ‘shallow or deficient affect,’ the majority of the seven children who met criteria for the LPE specifier were rated a ‘2’, meaning their behaviour was believed to be very descriptive on these three symptoms. No children meeting the criteria for LPE specifier scored ‘0’ for ‘lack of remorse or guilt’ or ‘callous-lack of empathy’ and only one of these children scored ‘0’ for ‘shallow or deficient affect.’ In contrast, the ratings for ‘unconcerned about performance’ did not seem to differ between children who met the criteria for the LPE specifier and those who did not.

### Do CAPE 1.1 ratings of CU traits relate to psychopathic traits, greater risk of violence and offending, and symptoms of internalizing and externalizing behaviour problems?

Table 1 notes the results of Spearman’s correlations examining the associations of the validation measures (questionnaires and case file records) with CAPE 1.1 ratings (number of symptoms rated 2). Spearman’s rho was used because of the non-parametric nature of many of the measures used. CAPE 1.1 scores were significantly correlated with CU traits (when measured by parent report only), psychopathic traits, ratings of violence from their case files, and the negative impact of their mental health symptoms on their daily living. There were no associations found between the CAPE 1.1 and externalizing/internalizing behaviour regardless of reporter.

**Table 1.** Spearman correlations among study measures. (Note: CPTI= Child Problematic Traits Inventory, GD= Grandiosity/deception, CU= Callous/unemotional, INS=Impulsivity/need-for-stimulation, UNSW= University of New South Wales, Child= Child-report, Parent= Parent-report, CAPE= Clinical Assessment of Prosocial Emotions).

	CAPE_ cat	Parent Internalizing	Parent Externalizing	Child Internalizing	Child Externalizing	Impact	cpti_gd	cpti_cu	cpti_ins	cpti_tot	UNSW CU parent	UNSW CU child	YOS	Violence
CAPE_cat	-													
Parent Internalizing	0.169	-												
Parent Externalizing	0.432 *	0.475 **	-											
Child Internalizing	-0.143	-0.099	-0.04	-										
Child Externalizing	0.046	0.117	0.305	0.066	-									
Impact	0.419 *	0.463 **	0.504 **	-0.002	0.356	-								
cpti_gd	0.52 **	0.406 *	0.748 ***	-0.103	0.232	0.42 *	-							
cpti_cu	0.614 **	0.521 **	0.616 ***	-0.231	0.211	0.501 **	0.68 ***	-						
cpti_ins	0.338 *	0.364 *	0.755 ***	-0.1	0.47 *	0.361 *	0.67 ***	0.549 ***	-					
cpti_tot	0.584 **	0.5 **	0.8 ***	-0.149	0.343	0.487 **	0.86 ***	0.849 ***	0.862 ***	-				
UNSW CU parent rating	0.539 *	0.585 ***	0.697 ***	-0.274	-0.066	0.443 **	0.71 ***	0.826 ***	0.535 **	0.813 ***	-			
UNSW CU child rating	0.163 **	-0.272	-0.123	-0.262	0.106	-0.14	0.01	-0.12	-0.033	-0.001	0.014	-		
YOS	0.053	-0.007	0.147	-0.305	0.192	0.062	0.18	0.243	0.426 *	0.382 *	0.183	0.016	-	
Violence	0.411 *	0.112	0.145	-0.433 *	0.208	0.06	0.3	0.383 *	0.264	0.409 *	0.335	0.411	0.289	-

Note. * p < .05, ** p < .01, *** p < .001

### Do children who meet the LPE specifier according to the CAPE 1.1 show greater psychopathic traits, greater risk of violence and offending, and symptoms of internalizing and externalizing behaviour problems?

**Figure 4.**
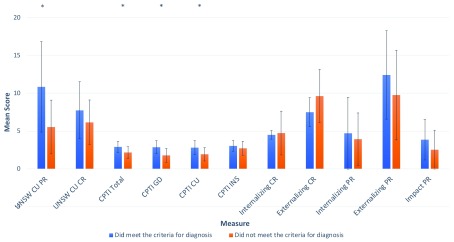
Mean scores (with SD bars) on validation measures by Limited Prosocial Emotions diagnostic status using the CAPE (Note: CPTI= Child Problematic Traits Inventory, GD= Grandiosity/deception, CU= Callous/unemotional, INS=Impulsivity/need-for-stimulation, UNSW= University of New South Wales, CR= Child-report, PR= Parent-report).

The final test of validity focused on whether the children who met criteria for the LPE specifier (n =7) would be different from those who did not (n = 27) on the various measures of problem behaviour and questionnaire measures of psychopathic traits. Of note, no girls met criteria for the LPE specifier. The mean scores and standard deviations for the parent and child report measures across the two groups are shown in Figure 4. Levene’s test of equality of variance was nonsignificant except for child reported internalizing behaviour, so we used Student’s t-tests except in that case. Those children diagnosed with the LPE specifier were found to have higher CU traits according to the UNSW parent report ( *t*(31)= -3.02, *p* = 0.005, 95%CI = -8.91, -1.72, *d* = -1.29) and higher psychopathic traits ( *t*(32)= -2.32, *p* = 0.027, 95%CI = -1.39, -0.09, *d* = -0.99) according to the CPTI total – specifically, they were higher on the grandiosity/deception subscale ( *t*(32)= -2.84, *p* = 0.008, 95%CI = -1.87, -0.31, *d* = -1.20) and the CU subscale ( *t*(32)= -2.32, *p* = 0.027, 95%CI = -1.65, -0.11, *d* = -0.99). The relatively small confidence intervals show the reliability of the estimation procedure used here have value. However, the two groups did not differ on the impulsivity/need for stimulation subscale of the CPTI ( *t*(32)= -0.84, *p =* 0.408, 95%CI = -1.09, 0.45, *d =* -0.36) or externalizing behaviours, either reported by parents or children ( *t*(31)= -1.06, *p =* 0.299, 95%CI = -7.80, 2.48, *d =* -0.45 ; *t*(21)= 1.16, *p =* 0.260, 95%CI = -1.70, 5.96, *d =* 0.64, respectively). The externalizing results are notable because the confidence intervals were large. Thus, power may be too low in the present study to yield reliable estimates of the true parameters. With regard to case file records, children meeting the criteria for the LPE specifier did not differ, from those not meeting criteria, on risk for violence or for contact with young offending services (X ^2^ (1, N = 32) = 1.52, p = .217, OR = 3.00, 95%CI = 0.50, 18.0; X ^2^ (1, N = 32) = 0.96, p = 0.327, OR = 2.38, 95%CI = 0.41, 13.7, respectively). Again, these confidence intervals are very large the reliability of the estimates are in question. The large confidence intervals are a sign that repeated samples are needed in future investigations.

## Discussion

In a sample of difficult-to-engage families, we showed that clinician ratings of CU traits using the CAPE 1.1 were associated with parent ratings of CU traits, psychopathic traits, and externalising behaviours. CAPE 1.1 ratings were also associated with risk for violence obtained from case files. These findings provide preliminary evidence for the validity of the CAPE 1.1 as a measure of CU traits, assessed in a way that is consistent with the Limited Prosocial Emotions (LPE) specifier for the diagnosis of Conduct Disorder included in the DSM-5 and ICD-11.

Since the CAPE 1.1 and UNSW CU traits measure are both derived from the ASPD and are therefore based on the same historical items, it is possible that they may use similarly worded items. However, as the CPTI was created differently, significant association between the CAPE 1.1 scores and the CU subscale of the CPTI suggests that findings do not solely rely on the validity between similarly worded items.

Using the diagnostic cut-off specified by the CAPE 1.1, we found that 21% of the target children met criteria for the LPE specifier, which is commensurate with estimates from prior research using detained ^48, 49^, clinic-referred ^50^, and high-risk ^51^ samples. When using this cut-off, those who scored above the diagnostic threshold were higher on parent ratings of CU traits and also ratings of grandiosity and deceptiveness, which are part of the interpersonal psychopathic traits. However, this group did not differ on the other measures of externalizing behaviour or risk for violence, which is similar to recent research that found the CAPE did not necessarily distinguish different offenders ^52^. There are two possible reasons for this finding. First, in the present study, the cut-off led to only 7 children in the sample meeting the diagnostic criteria, leading to very low power for detecting group differences, as evidenced by the large confidence intervals with some of the measures. Second, the use of a high-risk sample meant that even those who were not elevated on CU traits likely showed significant problems in adjustment. Thus, while the CAPE 1.1 may show prospective effects on delinquent behaviour or risky decision making within highly antisocial samples, this may not be apparent in cross-section research when the sample was selected for problem behaviour ^52, 53^.

The findings provide preliminary evidence for the diagnostic cut-off specified in the CAPE 1.1 manual as an appropriate measure to designate when the level of these traits warrants a diagnosis. CAPE 1.1 ratings using the sum of items scored at the maximum of “2” were significantly correlated with ratings of violence. This signifies that the prototypical presentation of low empathy, lack of guilt, lack of concern and shallow affect was associated with greater problems in adjustment across reporter-based measures but also case file records. Additionally, the greater number of areas where children showed this prototypicality were even more related to maladjustment. Further research with larger samples and with more diversity of problem behaviours is required to determine if the diagnostic cut-off is useful for this tool.

Importantly, the CAPE 1.1 provides a structured method for making clinical decisions, making it potentially useful for clinical settings that require more in-depth assessments than reliance simply on scores from rating scales. Clinical uses will become increasingly common now that CU traits are included in the diagnostic classification systems used globally. Further, as interventions are developed and tested to specifically target the needs of children and adolescents with elevated CU traits ^54^, their success will rely on adequate assessment, especially in samples who experience social disadvantage, behaviour problems, poor school attendance, and who show behaviours that are generally difficult to assess. Further, in such samples, clinical judgement will be important to ensure that reporters understand the questions and are able to provide information in a way that is appropriate for their cultural and educational background. Specifically, the CAPE 1.1 requires clinicians to gain examples from the informants in their own words, to ensure that the questions are understood and answered in the way that is intended.

Given that the CAPE 1.1 only leads to the rating of four items, it was somewhat surprising that they still formed a relatively internally consistent scale, which is similar to other more recent research ^52^. This finding suggests that scores from the CAPE 1.1 can be used as a continuous measure of CU traits as well. Further, three of the four items differentiated those who scored above the diagnostic criterion from those who did not. Specifically, the frequency at which the item “unconcerned about performance” was rated as being at the symptom level (i.e., a score of 2 or “very descriptive”) was similar for children who did and did not meet the diagnostic criteria for LPE. The small sample leading to a limited number of children meeting criteria for the specifier, again suggests that this finding should be replicated in other samples. However, it does point to the need to evaluate the relative utility of the symptoms, both in their sensitivity and in their specificity, used to define the LPE specifier ^50^.

These results need to be interpreted in light of several limitations. First, as noted previously, the sample size led to a very small number of children meeting the criteria for the LPE specifier and thus, there was limited power of the tests comparing those meeting and those not meeting the diagnostic threshold for the LPE specifier. The small sample also prevented us from testing potential moderators of the validity of CAPE 1.1. Of particular note, we could not test potential differences that might have been found in the validity of the CAPE 1.1 across age or sex of the child. Second, we only used parent and child reports from semi-structured interviews to score the CAPE 1.1; we did not have access to teachers as potential informants. Also, because we wanted to use behaviour ratings and case files to test the validity of the CAPE 1.1 scores, clinicians were not allowed to use this information in their ratings. Thus, clinicians were not able to use “all sources of available information”, as recommended by the CAPE 1.1 manual ^32^. Third, although the researchers carrying out the CAPE 1.1 had a sufficient level of training to do so (as previously outlined), they would not meet the criteria to make clinical decisions from its use relative to what is recommended in the manual. However, as the CAPE 1.1 was carried out solely for research purposes, rather than diagnostic purposes, that level of training was not necessary. Fourth, because of the training required and the method of obtaining information that relies on collecting multiple sources of information, the CAPE 1.1 is a much more time consuming and expensive method for assessing CU traits when compared to behaviour rating scales. While we have argued that this could be beneficial for many clinical uses, it will be important for future research to test whether the scores from the CAPE 1.1 provide important information over and above that provided by rating scales that would justify the cost.

Yet, there are notable strengths to using a semi-structured interview like the CAPE 1.1 for CU traits. For one, it is commonly observed in parent training programs that over the course of managing child behaviour problems, parents tend to become frustrated and may make global, dispositional, and sweeping attributions of their child’s challenging behaviour ^55^. The parent’s aim to make sense of their child’s challenging behaviour may evolve to protect them: for example, they may start to think “it’s my child’s naughty disposition, which has nothing to do with me”. Since parental ratings are often used in clinical interviews and assessments, it may be that their ratings of callous-unemotional traits are contaminated by dispositional attributions that arise out of their need to explain their child’s externalizing behaviour. However ^55^, Sawrikar *et al.* showed that dispositional ratings relate uniquely to parental feelings in comparison to CU traits, so these are separable. When conducting the assessment with the CAPE, parents are asked to describe the different contexts in which children may have shown cruelty or callousness. These types of follow-up questions test the when, where, and with whom, hopefully eliminating the ‘halo effect’ or the ‘horn effect’ where negative behaviour stains a child as ‘naughty’. They also divulge the contextual nature of behaving callously sometimes, which is patently different from a stable and consistent presentation of limited prosocial emotion. Additionally, clinicians are able to use their knowledge of typical development to query if the behaviour is part of maturational processes.

In sum, the CAPE 1.1 shows promise as a method for assessing CU traits in a way that is a) consistent with the diagnostic criteria for the LPE specifier and b) useful for making complex clinical decisions. Further, this promise was demonstrated in a sample of hard-to-reach families for whom clinical decisions may be difficult to make through other means. As a result, the CAPE 1.1 could provide a method for making important clinical decisions for children and adolescents who are at risk for a particularly severe and chronic pattern of conduct problems, for whom careful treatment planning is essential.

## Data availability

### Underlying data

Figshare: Centifanti TF target-child dataset for limited prosocial emotions using CAPE. https://doi.org/10.6084/m9.figshare.8300297 ^56^

This project contains the following underlying data:

- TF open data.csv (CAPE data for target children for whom the CAPE was completed. Sibling and family level data were removed for privacy)
- variable names.txt (Codebook for underlying data)

Data are available under the terms of the Creative Commons Attribution 4.0 International license (CC-BY 4.0).

### Extended data

Clinical Assessment of Prosocial Emotions, Version 1.1 (CAPE 1.1; ^32^): https://sites01.lsu.edu/faculty/pfricklab/cape/

Child Problematic Traits Inventory (CPTI; ^37, 38^): https://www.oru.se/english/research/research-environments/hs/caps/cpti/

Strengths and Difficulties Questionnaire (SDQ; ^39^): http://www.sdqinfo.com/

University of New South Wales (UNSW) Callous-Unemotional (CU) Traits ^43^: https://psycnet.apa.org/fulltext/2005-06517-003.pdf
