# Supplementary material for: CAPE for measuring callous-unemotional traits in disadvantaged families: a cross-sectional validation study
Source: F1000Res. 2020 Feb 27;8:1027. Originally published 2019 Jul 9. [Version 2] doi: 10.12688/f1000research.19605.2 (PMC7059787; doi:10.12688/f1000research.19605.2)
Supplement: Peer review history

# Peer review: Reviewer response for version 2

**DOI:** 10.5256/f1000research.24911.r60645

**Published:** 2020-03-18

**Authors:** Giorgos Georgiou

**Affiliations:** 1 Department of Social and Behavioral Sciences, European University Cyprus, Nicosia, Cyprus

Thanks for taking the time to reply and revised the manuscript. I read all the comments and I am satisfied with replies and changes. Since there is a revision of the paper based on reviewers comments I agree and recommend the indexing of this research.

I confirm that I have read this submission and believe that I have an appropriate level of expertise to confirm that it is of an acceptable scientific standard.

# Peer review: Reviewer response for version 2

**DOI:** 10.5256/f1000research.24911.r60643

**Published:** 2020-03-16

**Authors:** Timothy R. Stickle

**Affiliations:** 1 Department of Psychological Science, University of Vermont, Burlington, VT, USA

The authors have done a good job addressing concerns raised in initial reviews. This manuscript makes a nice contribution to the literature.

I confirm that I have read this submission and believe that I have an appropriate level of expertise to confirm that it is of an acceptable scientific standard.

# Peer review: Reviewer response for version 2

**DOI:** 10.5256/f1000research.24911.r60644

**Published:** 2020-03-05

**Authors:** Stuart F. White

**Affiliations:** 1 Center for Neurobehavioral Research (CNR), Boys Town National Research Hospital, Boys Town, NE, USA

The authors have addressed my concerns and put out a very nice contribution to the literature.

I confirm that I have read this submission and believe that I have an appropriate level of expertise to confirm that it is of an acceptable scientific standard.

# Peer review: Reviewer response for version 1

**DOI:** 10.5256/f1000research.21496.r56052

**Published:** 2019-11-15

**Authors:** Timothy R. Stickle

**Affiliations:** 1 Department of Psychological Science, University of Vermont, Burlington, VT, USA

This manuscript reports on a study of CAPE assessment for the limited prosocial emotions specifier to DSM-5 conduct disorder in a high-risk sample. Results make a contribution in that they provide support for the construct validity of the CAPE. Evidence for clinical utility is equivocal. There are a few areas for clarification that would help to better understand aspects of the study and its results. 

 Method/Sample

 The manuscript notes different age ranges. If I understand correctly the target children ranged in age from 5-18 years and that all children ranged in age from 3-19 years. I think it would help the reader to explicitly denote these age ranges in terms of target and comparison children (or some other fashion). Please comment on whether we should we expect the LPE specifier and thus the CAPE to perform similarly across this wide developmental range? 

 The total sample of children appears to be N = 85. This sample size should be stated explicitly as the manuscript states it somewhat indirectly in terms of describing a breakdown of 50 non-target and 35 target children when describing group characteristics. 

 Given that there are 34 families and, if I understood correctly, 85 children, ratings of children in the same family by the same parent are nested and so non-independent. The nesting calls into question the validity of p-values derived from tests of statistical significance assuming independence of observation. Some way of addressing this concern should be included. 

 An additional statistical concern, is low power, as acknowledged by the authors as a caveat to interpreting results. A formal power analysis should be included. This information will help the reader to better evaluate both significant and non-significant findings in terms of effect size and sample size. 

 Conclusions/Discussion

 The hypothesized utility of the LPE specifier is to identify children at higher risk for severe and persistent conduct problems and antisocial behavior. 

 The authors should address the utility of LPE/CAPE, given that no higher risk youth were identified in this sample. Some specific factors to address: The lack of identifying such a subgroup may be entirely due to insufficient statistical power. As noted above, asserting that low power led to this result requires a power analysis. It may also be that elevated risk may be evident over time rather than cross-sectionally.

I confirm that I have read this submission and believe that I have an appropriate level of expertise to confirm that it is of an acceptable scientific standard, however I have significant reservations, as outlined above.

# Response

**Published:** 2020-02-23

**Authors:** Luna Centifanti

**Affiliations:** University of Liverpool

Thank-you for the opportunity to revise and resubmit our paper to F1000Research. We are very glad to hear that there was interest in our manuscript and an appreciation for the contribution made with regards to providing support for the utility and validity of the CAPE. We would like to thank the three reviewers for their insightful and constructive comments on the first submission. Below we have responded to their comments and hope to have addressed the concerns adequately.

**Reviewer 3**

Method/Sample

- **The manuscript notes different age ranges. If I understand correctly the target children ranged in age from 5-18 years and that all children ranged in age from 3-19 years. I think it would help the reader to explicitly denote these age ranges in terms of target and comparison children (or some other fashion). Please comment on whether we should we expect the LPE specifier and thus the CAPE to perform similarly across this wide developmental range? **

This point was also raised by the second reviewer and we recognise that the information given is confusing. The first age range of 3-19 was descriptive of all children under the scheme but not all children were included in the study, therefore, we have removed this information to make it clearer.

We have also included the below information in the Method section to address concerns regarding the use of the CAPE across a wide developmental range.

*The manual specifies that raters use their developmental psychology training to ensure that symptoms are rated with regard to what is typical behaviour for a child based on their age. Both raters had sufficient training in developmental psychology and the assessment of callous-unemotional traits to assess children as young as 2-3 years of age (Waller et al., 2016).*

- **The total sample of children appears to be N = 85. This sample size should be stated explicitly as the manuscript states it somewhat indirectly in terms of describing a breakdown of 50 non-target and 35 target children when describing group characteristics. ** 

Thank-you for this suggestion. We have added the below to make this clearer.

*One of the target children was selected at random from the family that had two target children. Therefore, the total sample of children (N=84), consisted of 34 target children and 50 non-target children.*

- **Given that there are 34 families and, if I understood correctly, 85 children, ratings of children in the same family by the same parent are nested and so non-independent. The nesting calls into question the validity of p-values derived from tests of statistical significance assuming independence of observation. Some way of addressing this concern should be included. **

We have made the decision to remove the analyses for the part of the assessment that includes the non-target children - outlined in response to [Reviewer 1’s] comment 2 (a). Therefore this is no longer an issue.

- **An additional statistical concern, is low power, as acknowledged by the authors as a caveat to interpreting results. A formal power analysis should be included. This information will help the reader to better evaluate both significant and non-significant findings in terms of effect size and sample size. **

To address this we have added information about the relevant confidence intervals to the results section. Most confidence intervals were large, indicating that our estimations could be improved by a larger sample – which is something we later acknowledge in the discussion.

Conclusions/Discussion

- **The hypothesized utility of the LPE specifier is to identify children at higher risk for severe and persistent conduct problems and antisocial behavior. **
**The authors should address the utility of LPE/CAPE, given that no higher risk youth were identified in this sample. Some specific factors to address: The lack of identifying such a subgroup may be entirely due to insufficient statistical power. As noted above, asserting that low power led to this result requires a power analysis. It may also be that elevated risk may be evident over time rather than cross-sectionally.**

In response to Reviewer 1, we added some discussion of the clinical utility of the CAPE in the Discussion section. We also added more discussion around power analyses and whether prospective designs might be useful. In addition, two publications have since been published so we compare our findings about high-risk youth to their studies of clinic-referred and offending youth samples.

# Peer review: Reviewer response for version 1

**DOI:** 10.5256/f1000research.21496.r56051

**Published:** 2019-11-07

**Authors:** Giorgos Georgiou

**Affiliations:** 1 Department of Social and Behavioral Sciences, European University Cyprus, Nicosia, Cyprus

This interesting  manuscript investigates the CAPE 1.1 assessment in a difficult to access population range from 3 - 19 years old. While there is some indication regarding utility and validity of the CAPE 1.1, the impact of the findings is diminished by the small sample size and some minor issues.

 Introduction: 

- 1. Authors provide very detailed information regarding CAPE 1.1 and also the aim of the study. However, since authors investigated the difference between non-target with target children on several measures, something that is not mentioned in the introduction, they should revised aim section by adding this information.
- 2. LPE specifier is part of the diagnosis of Conduct disorder. In the current paper it is mentioned that participants come from high-risk, difficult to access families. However it is useful to inform readers if those children exhibit symptoms that lead to the diagnosis of CD or any other comorbidity. 

Method:

- 3. In the participants section it is mentioned that children's ages ranged between 3 -19 years old. In the next paragraph the descriptive information of target children are not consisted (it is mentioned a range of 5-18 years old). It should be clarified if target children are the actual participants and if this is the case then why there are different descriptive statistics and range of age.
- 4. It is mentioned that 6 families were deemed unsuitable or declined. Authors should provide the reasons that lead to this decision (not in details).
- 5. In CAPE 1.1 description it is stated that a child with a diagnosis of ASD was included in the study. I am not convinced that a child with autism can participate in the current study. Despite their similarities in behavior and in empathy difficulties, ASD and CU traits have different underlying mechanisms. Thus, authors should not use the data of this participant or justify the decision of including them.

Results:

- 6. A descriptive table will help readers to have a more clear idea of all basic demographics information.
- 7. In figure 1, authors provide very useful information regarding the differences between target and non-target children. However, it is not mentioned in the introduction and method section that authors will investigate this. Thus, it should be stated as an aim of the study and also in both measures and participants section.
- 8. Figure 4 (correlations) is very difficult to follow

Discussion:

- 9. Discussion is well written and the limitation section discuss all issues that are raised (both in method and interpretation). However authors again do not mention anything about the non-target children.

I confirm that I have read this submission and believe that I have an appropriate level of expertise to confirm that it is of an acceptable scientific standard, however I have significant reservations, as outlined above.

# Response

**Published:** 2020-02-23

**Authors:** Luna Centifanti

**Affiliations:** University of Liverpool

Thank-you for the opportunity to revise and resubmit our paper to F1000Research. We are very glad to hear that there was interest in our manuscript and an appreciation for the contribution made with regards to providing support for the utility and validity of the CAPE. We would like to thank the three reviewers for their insightful and constructive comments on the first submission. Below we have responded to their comments and hope to have addressed the concerns adequately.

**Reviewer 2**

Introduction

- **Authors provide very detailed information regarding CAPE 1.1 and also the aim of the study. However, since authors investigated the difference between non-target with target children on several measures, something that is not mentioned in the introduction, they should revise the aim section by adding this information.**

We agree with this point and have removed this part of the assessment. This is outlined in response to [Reviewer 1’s] comment 2 (a).

- **LPE specifier is part of the diagnosis of Conduct disorder. In the current paper it is mentioned that participants come from high-risk, difficult to access families. However, it is useful to inform readers if those children exhibit symptoms that lead to the diagnosis of CD or any other comorbidity. **

Local authorities identified families to take part in the government schemes. As researchers were not involved in this process, we cannot comment on whether the children of those families exhibited symptoms. However, we do acknowledge that children exhibiting symptoms of conduct problems often come from high-risk, difficult to access families.

*Children who show serious conduct problems tend to come from high-risk backgrounds involving disorganized, unmotivated or disadvantaged families.*

Method

- **In the participants section it is mentioned that children's ages ranged between 3 -19 years old. In the next paragraph the descriptive information of target children are not consisted (it is mentioned a range of 5-18 years old). It should be clarified if target children are the actual participants and if this is the case then why there are different descriptive statistics and range of age.**

Thanks for noticing this. The first age range of 3-19 was descriptive of all children under the scheme but not all children were included in the study, therefore, we have removed this information to make it clearer.

- **It is mentioned that 6 families were deemed unsuitable or declined. Authors should provide the reasons that lead to this decision (not in details).**

Thank-you for pointing out that this was not clear, we have added the below information for clarification.

*These were unsuitable because either the caseworkers were themselves afraid of violence or the families were already non-compliant with the caseworkers efforts at intervention.*

- **In CAPE 1.1 description it is stated that a child with a diagnosis of ASD was included in the study. I am not convinced that a child with autism can participate in the current study. Despite their similarities in behavior and in empathy difficulties, ASD and CU traits have different underlying mechanisms. Thus, authors should not use the data of this participant or justify the decision of including them.**

Thank-you for this valuable input, we have now added in the below information to justify our decision for including children with ASD.

*We decided to do assessments using the CAPE regardless of potential diagnoses of Autism Spectrum Disorder (ASD), because research shows that some children have a ‘double hit’ of ASD and callous-unemotional traits (Pasalich et al., 2014) and the modest phenotypic overlap observed in social-emotional deficits is largely explained by shared genetic variance (O’Nions et al., 2015).*

Results

- **A descriptive table will help readers to have a more clear idea of all basic demographics information.**

We agree with this comment and have therefore added a figure (Figure 1) to make the demographic information clearer.

- **In figure 1, authors provide very useful information regarding the differences between target and non-target children. However, it is not mentioned in the introduction and method section that authors will investigate this. Thus, it should be stated as an aim of the study and also in both measures and participants section.**

To ensure the focus remains on the validation of the CAPE we have chosen to remove the assessment of the non-target youth. This is outlined in response to [Reviewer 1’s] comment 2 (a).

- **Figure 4 (correlations) is very difficult to follow**

In light of this comment and [Reviewer 1’s] comment 6 (b), we have now presented the information in a correlation table.

Discussion

- **Discussion is well written, and the limitation section discuss all issues that are raised (both in method and interpretation). However, authors again do not mention anything about the non-target children.**

Thank-you for such positive feedback on our discussion. We have now removed the assessment of non-target children, as we agree the purpose for inclusion was not clear. This is outlined in response to [Reviewer 1’s] comment 1 (a).

# Peer review: Reviewer response for version 1

**DOI:** 10.5256/f1000research.21496.r54368

**Published:** 2019-10-01

**Authors:** Stuart F. White

**Affiliations:** 1 Center for Neurobehavioral Research (CNR), Boys Town National Research Hospital, Boys Town, NE, USA

The current manuscript investigates the CAPE assessment in a very difficult to access population. While there is some indication of the validity and utility of the CAPE from these data, the impact of the findings is diminished by the small sample size.

 Introduction

1. Please reference both the “Troubled Families Programme,” and the “Family Intervention Project” in the introduction, or just say “drawn from government intervention programs” or similar. It was slightly confusing tracking back to the introduction from the methods when I expected only one program and two were described.
2. Please discuss why the non-target youth were assessed in this study in the introduction.

Methods/Results

1. The authors write, “no sample size calculation was performed.” I suspect that the authors mean that no power analysis was conducted to inform sample size. Please be clear.
2. The level of detail regarding the clinical training of the interviewers seems a bit excessive. Something along the lines of “the interviewers had sufficient clinical training and research experience to conduct the interviews, though were not registered clinicians” should suffice. That the interviews were not conducted by the typical clinician is a weakness that should be discussed later in the paper.
3. The comparison between target and non-target youth comes a bit from no-where. This analysis is not well set-up in the introduction. Please make it clear why this analysis is conducted in the introduction.
4. In Figures 1 & 5, please report standard error bars, as opposed to standard deviation.
5. The sentence “There were no associations found between the CAPE 1.1 and externalizing/internalizing behavior regardless of reporter, although the relation with externalizing behaviour (as reported by the parent) was moderate” is unclear. If there was no association, how can the relationship be moderate?
6. Figure 4 is very difficult to read - it is a jumble. Perhaps indicate via color-code specific ranges of correlation/significant correlations. The specific numbers and the crossing out of those numbers is less than ideal.

Discussion

1. The non-target children are not mentioned in the discussion. They either need to be incorporated more fully into the paper or removed from the paper.
2. I think that the authors need to spend more time in the discussion making the case that these data are able to help clinicians evaluate the CAPE. The authors did an admirable job of accessing a difficult population, but the reality is that the numbers are so low, it’s hard to interpret the data. This is especially the case since the CAPE wasn’t used entirely in a manner consistent with recommended clinical use. Why would a clinician, after reading this paper, make a decision one way or the other about using the CAPE in clinical practice? The discussion needs to take this criticism on directly and forcefully. 

Minor points:

1. The following sentence is clunky: “However, negatively worded items can sometimes be difficult to understand and put a strain on a child’s verbal abilities, which are often found to be deficient in children with conduct problems, anyway.” Perhaps revise?
2. “No other exclusion criteria were necessary” is an odd way to write this. Please be clear that no other exclusion criteria were employed.

I confirm that I have read this submission and believe that I have an appropriate level of expertise to confirm that it is of an acceptable scientific standard, however I have significant reservations, as outlined above.

# Response

**Published:** 2020-02-23

**Authors:** Luna Centifanti

**Affiliations:** University of Liverpool

Thank-you for the opportunity to revise and resubmit our paper to F1000Research. We are very glad to hear that there was interest in our manuscript and an appreciation for the contribution made with regards to providing support for the utility and validity of the CAPE. We would like to thank the three reviewers for their insightful and constructive comments on the first submission. Below we have responded to their comments and hope to have addressed the concerns adequately.

**Reviewer 1**

Introduction

- **(a) Please reference both the “Troubled Families Programme,” and the “Family Intervention Project” in the introduction, or just say “drawn from government intervention programs” or similar. It was slightly confusing tracking back to the introduction from the methods when I expected only one program and two were described.**

Thank-you for this comment, we agree with this point and have changed the introduction and the method section of the paper to make this clearer.

- **(a) Please discuss why the non-target youth were assessed in this study in the introduction.**

In light of the number of comments made surrounding the inclusion of the non-target youth, we have made the decision to remove this part of the assessment. As the paper is about validating the CAPE, we want to ensure this is where the focus remains.

Methods/Results

- **(b) The authors write, “no sample size calculation was performed.” I suspect that the authors mean that no power analysis was conducted to inform sample size. Please be clear.**

This is what was meant, so we have changed “no sample size calculation was performed” to “no power analysis was conducted to inform sample size,” as suggested.

- **(b) The level of detail regarding the clinical training of the interviewers seems a bit excessive. Something along the lines of “the interviewers had sufficient clinical training and research experience to conduct the interviews, though were not registered clinicians” should suffice. That the interviews were not conducted by the typical clinician is a weakness that should be discussed later in the paper.**

We included the specific qualifications of the interviewers for transparency but agree that this level of detail for the purposes of the paper is excessive. Therefore, we have cut down the information provided.

*The CAPE 1.1 was carried out by the first and fourth authors (LCMC and NDT), who despite not being registered clinicians had a sufficient level of qualification, clinical training and research experience to carry out the assessments.*

- **(b) The comparison between target and non-target youth comes a bit from no-where. This analysis is not well set-up in the introduction. Please make it clear why this analysis is conducted in the introduction.**

We agree with this point and have removed this part of the assessment. This is outlined in response to [Reviewer 1’s] comment 2 (a).

- **(b) In Figures 1 & 5, please report standard error bars, as opposed to standard deviation.**

Figure 1 has been removed from the paper as we have made the decision to remove this part of the assessment (see response to [Reviewer 1’s] comment 2a). Figure 5 is now referred to as Figure 4 and standard deviations have been changed to standard error bars.

- **(b) The sentence “There were no associations found between the CAPE 1.1 and externalizing/internalizing behavior regardless of reporter, although the relation with externalizing behaviour (as reported by the parent) was moderate” is unclear. If there was no association, how can the relationship be moderate?**

Thank-you for noticing this, this was in error and we have corrected it.

*CAPE 1.1 scores were significantly correlated with CU traits (when measured by parent report only), externalising behaviour (when measured by parent report only), psychopathic traits, ratings of violence from their case files, and the negative impact of their mental health symptoms on their daily living. There were no associations found between the CAPE 1.1 and internalizing behaviour regardless of reporter.*

- **(b) Figure 4 is very difficult to read - it is a jumble. Perhaps indicate via color-code specific ranges of correlation/significant correlations. The specific numbers and the crossing out of those numbers is less than ideal.**

To make the information easier to read, we have now presented this in a correlation table.

Discussion

- **(C) The non-target children are not mentioned in the discussion. They either need to be incorporated more fully into the paper or removed from the paper.**

We agree with this point and have removed this part of the assessment. This is outlined in response to [Reviewer 1’s] comment 2 (a).

- **(C) I think that the authors need to spend more time in the discussion making the case that these data are able to help clinicians evaluate the CAPE. The authors did an admirable job of accessing a difficult population, but the reality is that the numbers are so low, it’s hard to interpret the data. This is especially the case since the CAPE wasn’t used entirely in a manner consistent with recommended clinical use. Why would a clinician, after reading this paper, make a decision one way or the other about using the CAPE in clinical practice? The discussion needs to take this criticism on directly and forcefully. **

Thank-you for this comment. We have added a paragraph to the discussion to address why we think the CAPE would be useful in clinical practice.

Minor points:

- **(D) The following sentence is clunky: “However, negatively worded items can sometimes be difficult to understand and put a strain on a child’s verbal abilities, which are often found to be deficient in children with conduct problems, anyway.” Perhaps revise?**

We have taken some words out of this sentence in the hope that it is now easier to read.

*However, negatively worded items can be difficult to understand and put a strain on a child’s verbal abilities, which have been found to be deficient in children with conduct problems.*

- **(D) “No other exclusion criteria were necessary” is an odd way to write this. Please be clear that no other exclusion criteria were employed.**

Thank-you for noticing this. We have changed ‘no other exclusion criteria were necessary’ to *‘no other exclusion criteria were employed,’* as suggested.
